# Clusters formed by dumbbell-like one-patch particles confined in thin systems

**DOI:** 10.1038/s41598-021-97542-7

**Published:** 2021-09-10

**Authors:** Masahide Sato

**Affiliations:** Emerging Media Initiative, Kanazawa University, Kanazawa, 920-1192 Japan

**Keywords:** Colloids, Molecular self-assembly

## Abstract

Performing isothermal-isochoric Monte Carlo simulations, I examine the types of clusters that dumbbell-like one–patch particles form in thin space between two parallel walls, assuming that each particle is synthesized through the merging of two particles, one non-attracting and the other attracting for which, for example, the inter-particle interaction is approximated by the DLVO model . The shape of these dumbbell-like particles is controlled by the ratio of the diameters *q* of the two spherical particles and by the dimensionless distance *l* between these centers. Using a modified Kern–Frenkel potential, I examine the dependence of the cluster shape on *l* and *q*. Large island-like clusters are created when $q<1$. With increasing *q*, the clusters become chain-like . When *q* increases further, elongated clusters and regular polygonal clusters are created. In the simulations, the cluster shape becomes three-dimensional with increasing *l* because the thickness of the thin system increases proportionally to *l*.

## Introduction

Particles having patch areas in which properties are different from those of other surface areas are termed patchy particles. Many groups^1–24^ have synthesized patchy particles using different methods and examined the self-assemblies formed by patchy particles. Because patchy particles are promising building blocks for functional materials, efficient synthetic methods and properties of self-assemblies have been studied intensely. For example, triblock patchy particles having two patches on the polar positions^3–5,8,23,24^ have drawn much attention as building blocks for photonic crystals with a complete photonic band gap^25,26^. Whereas patchy particles used in experiments were not necessarily spherical^6,11,16^, the structures and cluster shapes examined in a theoretical study^27^ and simulations^28–41^ were mainly for spherical patchy particles.

In studies on non-spherical patchy particles, Monte Carlo simulations of dumbbell-like one-patch particles with a modified Kern–Frenkel potential were performed^42–44^, and the self-assemblies created by such particles were studied. It was shown that several types of clusters such as spherical micelles, elongated micelles, vesicles, and bilayers are created in three-dimensional systems^42,43^ by controlling the shape of the dumbbell-like one-patch particle. When the long axis of such particles is fixed within a flat plane, island-like clusters with voids, mesh-like clusters, and straight chain-like clusters are observed in addition to elongated clusters and isotropic clusters in two-dimensional systems^44^. In previous studies^42–44^, the attraction length in the modified Kern–Frenkel potential was set to be as long as the radius of the attractive sphere of the dumbbell-like particles. However, in several experiments^7,9^, the attraction length was revealed to be much smaller than the radius of patchy particles. Because the attraction length in the Kern–Frenkel potential^45^ affects the structure and shape of clusters, even for spherical patchy particles^36,46–48^, the shape of the clusters formed by dumbbell-like patchy particles probably depends on the attraction length as well.

**Figure 1:**
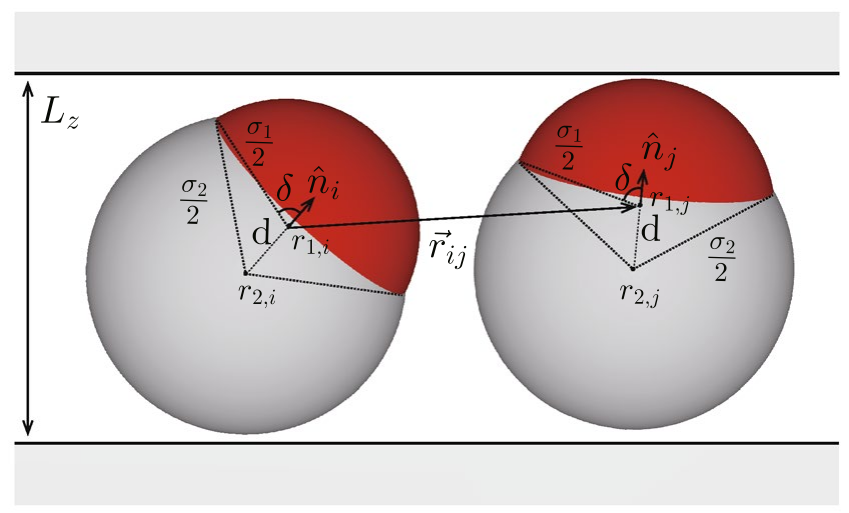
System used in simulations, where dumbbell-like one-patch particles are confined between two flat walls. The particles are synthesized by merging two spheres. Red and white regions represent attractive area and non-attractive area, respectively. The shape of particles is characterized by $q = \sigma _2/\sigma _1$ and $l=2d /(\sigma _1 + \sigma _2)$, where $\sigma _2$ and $\sigma _1$ denote the diameters of the non-attractive and attractive spheres, respectively, *d* the distance between the centers of the two spheres, and *l* the dimensionless distance scaled by average diameter. $\delta$ satisfies $\cos \delta = (\sigma _2^2 -\sigma _1^2 -4 d^2) /4d \sigma _1$ $=2l/(1-q)-(1+q) l^2.$ The particle shape is more dumbbell-like with increasing *d*. The unit vector directed from the center of the non-attractive sphere to that of the attractive sphere is denoted as $\varvec{{\hat{n}}}$. $\sigma _1$ is set to unit, and the *x*- and *y*-directions are set parallel to the walls, and the *z*-direction is set perpendicular to the walls, which are given by $z=0$ and $z=L_z$. With the distance between the two walls denoted by $L_z$, the lengths of the wall in the *x*- and *y*-directions, $L_x$ and $L_y$, are given by $L_x =L_y = \sqrt{vN/(\rho L_z)}$, where *v*, $\rho$, and *N* denote the volume of the dumbbell-like particle, the particle density, and the number of particles, respectively. The distance between the two walls $L_z$ is set to $1.1 [(\sigma _1 + \sigma _2)/2 + d ]$. Because $L_z$ is slightly longer than the long axis in the dumbbell-like patchy particles, the particles rotate three-dimensionally.

In this paper, I describe isothermal-isochoric simulations for dumbbell-like patchy particles in thinly confined systems as shown in Fig. 1, in which the attraction length of the modified Kern–Frenkel potential is set shorter than that in previous studies^42–44^. I examine how cluster types depend on the shapes of dumbbell-like patchy particles. In the simulations, the focus is on the formation of clusters in thin systems because films of high quality are required as substrates for colloidal epitaxy^39,49–51^. Compared with creating quality three-dimensional functional materials spontaneously in open three-dimensional spaces, creating the desired structures on substrates by epitaxial growth may be easier. The formation of two-dimensional materials and quasi two-dimensional materials is also a popular topic because thin sheet materials are useful in a broad range of applications such as photovoltaics, semiconductors, electrodes, water purification^52,53^. The types of the two-dimensional clusters and structures that are formed when the long axes of dumbbell-like particles are fixed in a flat plane have already been studied ^44^. Different clusters may be created if the dumbbell-like one-patch particles rotate freely three-dimensionally. Under this scenario, I investigate in the rest of this paper the formation of clusters and structures in thin systems. First, I show several typical snapshots of simulations. Then, I introduce four parameters and show how cluster shapes and structures formed by dumbbell-like patchy particles depend on particle shape.

## Results and discussions

Isothermal-isochoric Monte Carlo simulations, performed with number of particles $N=1024$ and the particle density $\rho =0.2$ in the system shown in Fig. 1, provided the dataset to examine how cluster shape depends on the shape of dumbbell-like particles.

**Figure 2:**
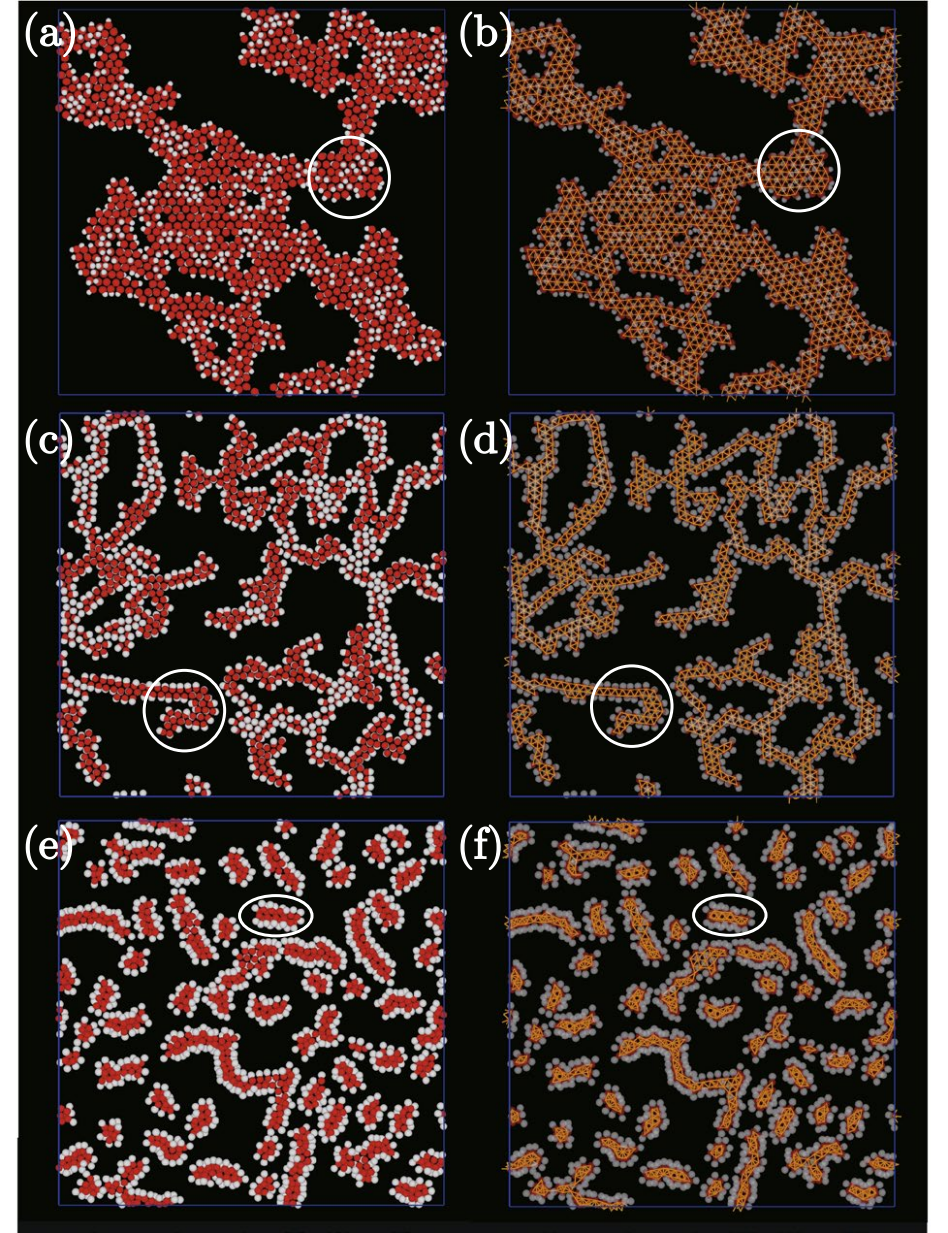
Typical snapshots of large clusters for $\epsilon /k_{\mathrm {B}}T=8.0$ as viewed from the *z*-direction. The parameter setting for (*l*, *q*) are (**a**) and (**b**) (0.70, 0.70), (**c**) and (**d**) (0.50, 1.05), and (**e**) and (**f**) (0.85, 1.05). Only particle positions are drawn in (**a**), (**c**), and (**e**). The white and red areas show the non-interacting and attracting areas, respectively. In (**b**), (**d**), and (**f**), in addition to the particle positions, attracting particles are connected by lines.

**Figure 3:**
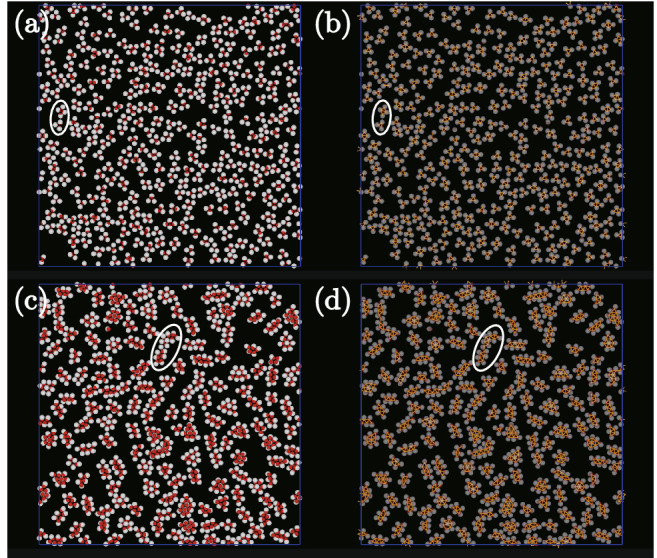
As for Fig. 2 but with (*l*, *q*) set to (0.20, 1.25) in (**a**) and (**b**), and (0.45, 1.25) in (**c**) and (**d**).

**Figure 4:**
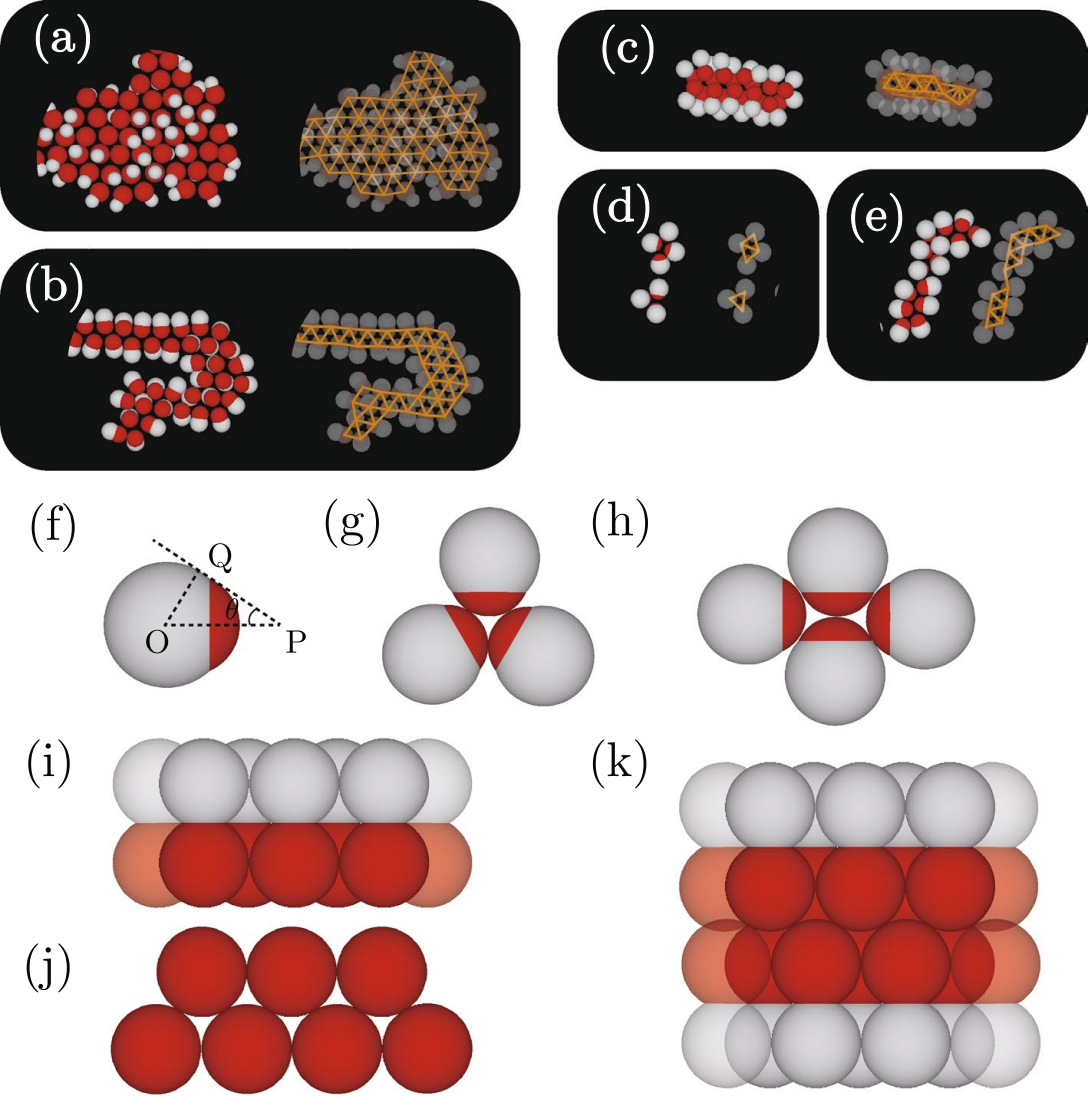
(**a**–**d**) Structures and clusters surrounded by circles or ovals in Figs. 2 and 3 . The settings for (*l*, *q*) are (a) (0.70, 0.70), (b) (0.50, 1.05), (**c**) (0.85, 1.05), (**d**) (0.20, 1.25), and (**e**) (0.45, 1.25). (**f**) Typical dumbbell-like one-patch particle for a small interaction part, (**g**) triangular trimer, and (**h**) square tetramer. (**i**) Top view and (**j**) side view of a three-dimensional single array for $q=1$, and (**k**) top view of the three-dimensional double chain-like structure formed by two single arrays.

### Typical snapshots for large clusters

Figures 2 and 3 show typical snapshots viewed from the *z*-direction. The temperature *T* satisfies $\epsilon /k_{\mathrm {B}}T=8.0$, where $k_{\mathrm {B}}$ is Boltzmann’s constant and $\epsilon$ denotes the attractive energy. The zoomed snapshots of the areas surrounded by white circles in each figure are shown in Fig. 4. One large island-like cluster is created when $(l,q)= (0.7, 0.7)$ (see Fig. 2a). Almost all the dumbbell-like patchy particles are connected and included in the island-like cluster. Because $\sigma _2$ is smaller than $\sigma _1$, the steric hindrance caused by the non-attractive region is weak when $\varvec{{\hat{n}}}$ of every particle is perpendicular to the walls. Whereas $\varvec{{\hat{n}}}$ is almost perpendicular to the walls for the most of particles inside the island-like cluster, $\varvec{{\hat{n}}}$ for particles located at the edge of the cluster fluctuate because the number of neighboring particles is small. Moreover, the binding of particles at the cluster edge is weak, and therefore the particles at the edge of the island-like cluster rotate easily under thermal fluctuations. In our previous study^44^, a square lattice with voids is created because the interaction length is set to $\sigma _1/2$, and particles at the diagonal positions can attract each other. However, the particles inside the large island-like cluster form a triangular lattice because the attraction length is so short that the particles at diagonal positions cannot interact with each other even if a square lattice is made (Figs. 2b and 4a). The attracting particles are most numerous in a triangular lattice when the interaction length is sufficiently short. With the number of neighbors being six, the energy change per particle is $3\epsilon$.

Creating large island-like clusters with increasing *q* or *l* becomes hard because steric hindrance arising from the non-attractive area in particles increases. The cluster changes from forming islands to forming chains (Fig. 2c and d). In these figures, several particles with six neighbors are seen in places. However, when one particle has six neighbors, $\varvec{{\hat{n}}}$ of the neighboring particles tilts from the *z*-direction (see Fig. 4b) because $\sigma _1$ is smaller than $\sigma _2$. Therefore, neighboring particles cannot have six connections. Steric hindrance incurred by the non-attractive area suppresses the creation of large island-like clusters, and two-dimensional chain-like clusters consisting of two arrays of dumbbell-like patchy particles form. The particles in perfectly straight chain-like clusters have four connecting neighbors, two neighbors in the same array and in the other array diagonally in front. The energy gain per particle by forming the chain-like clusters is $2 \epsilon$.

### Typical snapshots for small clusters

The shape of the dumbbell-like particle becomes more anisotropic with increasing *l*. The system width $L_z$ increases with increasing *l* because the system width is set so that the dumbbell-like one-patch particles are able to rotate easily in the thin systems. When $q=1$ and $l>\sqrt{3}/2$, creating three-dimensional arrays is possible if $\varvec{{\hat{n}}}$ of each dumbbell-like patchy particle is parallel to the *xy*-plane. Top and side views of a portion of the three-dimensional arrays are presented in Fig. 4i and j, respectively. When the two arrays attract each other and double chain-like clusters form, as in Fig. 4k, each particle in the double chain-like clusters attracts six other particles, their number being the same as the number of neighbors in an island-like cluster. The radius of the non-attractive area in the dumbbell-like patchy particles is larger than that of the attractive part for $q>1$. Through steric hindrance from the non-attractive sphere, the three-dimensional double chain-like clusters need to be curved (Fig. 4k). With a further decrease in *q*, the curved chain-like clusters are easily torn off creating three-dimensional elongated clusters (Figs. 2e, f and 4c). With *q* constant and *l* decreased, instead of the formation of three-dimensional elongated clusters, two-dimensional elongated clusters form (Figs. 3c, d, and 4e).

### Typical snapshots for regular clusters

Figure 3a and b show snapshots for small *l* and large *q*. Many small two-dimensional polygonal clusters, for example, triangular trimers (Fig. 4g) and rhomboidal tetramers (Fig. 4h) are created (Fig. 4d). The *q* and *l* condition for forming triangular trimers is easily estimated. Figure 4f shows the typical shape of a dumbbell-like one-patch particle for which $\varvec{{\hat{n}}}$ is parallel to the *xy*-plane; here O labels the center of the non-attractive sphere, and PQ the common tangent of the non-attractive sphere and the attractive sphere that determine angle $\theta = \angle$PQO satisfying $\sin \theta = (\sigma _2 -\sigma _1)/2d=(q-1)/l(q+1)$. The angle $\delta$ (see Fig. 1) needs to be larger than $30^\circ$ for dumbbell-like patchy particles to create triangular trimers and $\theta$ should be smaller than $60^\circ$ to avoid steric hindrance induced by the non-attractive sphere. The condition for $\theta$ is slightly stricter than that for $\delta$ when $q>1$. Therefore, to create triangular trimers, *l* and *q* need to satisfy $l> 2(q-1) /\sqrt{3}(q+1)$. With $q=1.25$, *l* needing to be larger than 0.128 to create triangular trimers, the condition for forming trimers indicated in Figs. 3c and 4d is satisfied. For rhomboidal tetramers, the criterion for avoiding steric hindrance is slightly more complicated to estimate, but that for $\delta$ is easier. Because $\delta$ is larger than $60^\circ$ to create rhomboidal tetramers, we obtain the inequality $l > (-1 + \sqrt{4 q^2-3})/2(1+q)$. Given *l* needs to be larger than 0.178 when $q=1.25$, the condition required in forming rhomboidal tetramers seen in Fig. 3 seems reasonable.

**Figure 5:**
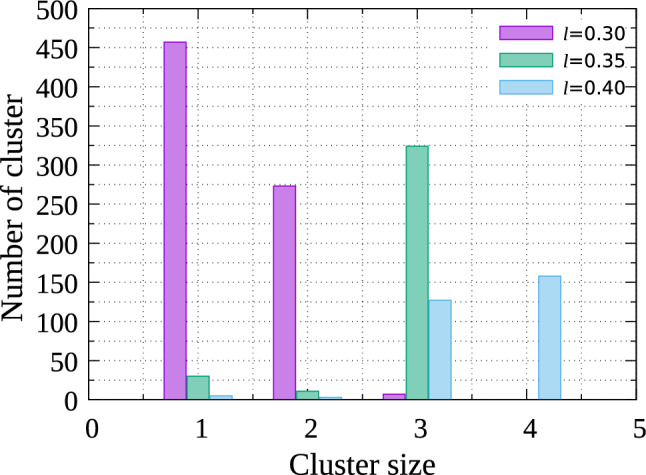
Distribution of cluster size for $(l, q)=(0.3, 1.6)$, (0.35, 1.6), and (0.4, 1.6) with $\epsilon /k_{\mathrm {B}}T=8.0$.

I also examine whether the formation of regular timers and tetramers obeys the criteria for another *q*. For $q=1.6$, the range of *l* values for which two-dimensional triangular timers and two-dimensional rhomboidal tetramers are created are estimated to $l> 0.27$ and $l> 0.33$, respectively. Figure 5 shows the cluster size distributions observed from simulations with $\epsilon /k_{\mathrm {B}}T=8.0$. When $l<0.30$, almost all particles are monomers although the formation of dimers is allowed, which is probably because the attractive area is too small to make stable dimers with this temperature. With $l=0.30$, many dimers are created although many monomers still remain. As expected from the criterion given by the inequality for the formation of trimers, a few trimers are also created. With $l=0.35$, the main clusters are trimers. Tetramers are not observed, although their formation is expected from the inequality for the formation of tetramers because *l* is very close to the formation threshold. When $l=0.4$, tetramers are created because *l* is above the threshold. Because the energy gain by the formation of two-dimensional tetramers is larger than that of two-dimensional trimers, tetramers are created in higher numbers than trimers.

### Classification of cluster types by order parameters

To classify cluster types systematically and to show how the cluster type depends on the particle shape quantitatively, four parameters $P_{\mathrm {s}}=\sum _{k}k N_k/N$, $P_{z}=\sum _{i}|n_{zi}|/N$, ${\mathcal {M}}= (N-N_1)^{-1} \sum _i \varvec{{\hat{n}}}_i \cdot (\varvec{r}_{\mathrm {c}1}-\varvec{r}_{1,i} )/|\varvec{r}_{\mathrm {c}1}-\varvec{r}_{1,i} |$, and $\sigma _z = \sqrt{N^{-1} \sum _i {z}_{1,i}^2 -\left( N^{-1} \sum _i z_{1,i} \right) ^2 }$ are introduced, in which $N_k$ denotes the number of clusters formed by *k* particles, $n_{zi}$ the *z*-component of $\varvec{{\hat{n}}}_i$, $N_1$ the number of monomers, $\varvec{r}_{1,i}$ the center of the attractive sphere of the *i*-th particle, $\varvec{r}_{\mathrm {c}1}$ the average position of attractive spheres for the cluster including the *i*-th particle, and ${z}_{1,i}$ is the z-coordinate of the center of the attractive sphere of the *i*-th particle. In the definition of ${\mathcal {M}}$, the summation does not include monomers.

**Figure 6:**
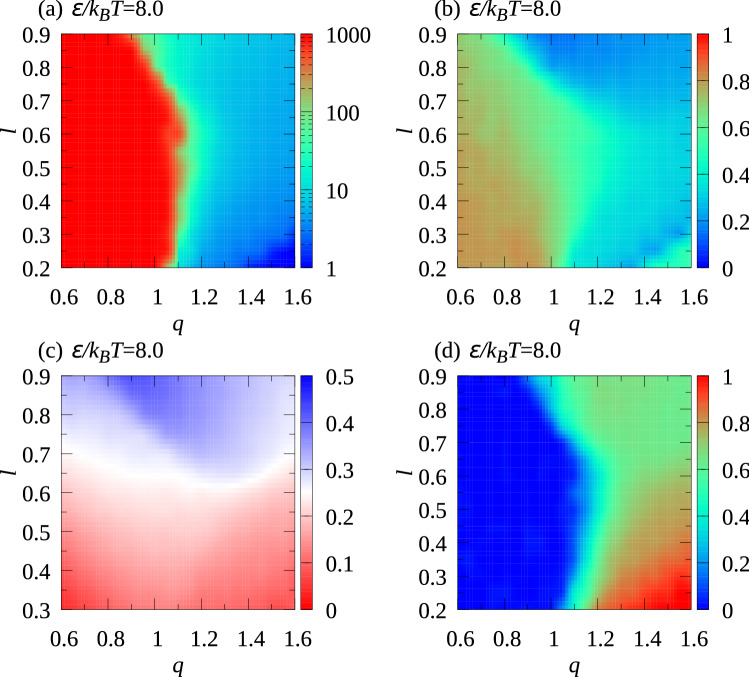
Dependence of (**a**) $P_{\mathrm {s}}$, (**b**) $P_z$, (**c**) $\sigma _z$, (**d**) ${\mathcal {M}}$ on *q* and *l* for $\epsilon /k_{\mathrm {B}}T=8.0$. (**e**) Top and (**f**) side views, and (**g**) top view of a double chain-like structure formed by two single chain-like clusters.

The *l* and *q* dependence of $P_{\mathrm {s}}$, $P_z$, $\sigma _z$, and ${\mathcal {M}}$ depend on *l* and *q* for $\epsilon /k_{\mathrm {B}}T=8.0$ were obtained (Fig. 6). $P_{\mathrm {s}}$ is found to be small with $q>1$ but suddenly increases around $q=1$ (see Fig. 6a). With $q<1$, $P_{\mathrm {s}}$ is over 1000, which means that almost all particles in the system are connected and one large cluster is created. Large $P_{\mathrm {s}}$ indicates the formation of large clusters, but I cannot identify whether the cluster type is large island-like or chain-like. From Fig. 2, $n_z$ is large for particles in island-like clusters but small for chain-like clusters. Therefore, $P_{z}$ is a useful parameter for determining the shape of large clusters. Because $P_z$ is large when both *q* and *l* are small (Fig. 6b), large island-like clusters are created in this parameter regime.

**Table 1:** Parameter values used to classify cluster types are $P_{\mathrm {s, c}}= 100$, $P_{z, {\mathrm {c}}}=0.65$, $\sigma _{z, {\mathrm {c}}}=0.25$, and ${\mathcal {M}}_{\mathrm {c}} =0.8$.

Structure and cluster shape	$P_{\mathrm {s}}$	$P_{z}$	$\sigma _z$	${{\mathcal {M}}}$
Two-dimensional island-like cluster	$> P_{\mathrm {s, c}}$	$> P_{z, {\mathrm {c}}}$	–	–
Two-dimensional chain-like cluster	$> P_{\mathrm {s, c}}$	$< P_{z, {\mathrm {c}}}$	–	–
Two-dimensional regular cluster	$< P_{\mathrm {s, c}}$	$< P_{z, {\mathrm {c}}}$	$< \sigma _{z, {\mathrm {c}}}$	$<{{\mathcal {M}}}_{\mathrm {c}}$
Two-dimensional elongated cluster	$< P_{\mathrm {s, c}}$	$< P_{z, {\mathrm {c}}}$	$< \sigma _{z, {\mathrm {c}}}$	$>{{\mathcal {M}}}_{\mathrm {c}}$
Three-dimensional elongated cluster	$< P_{\mathrm {s, c}}$	$< P_{z, {\mathrm {c}}}$	$> \sigma _{z, {\mathrm {c}}}$	$>{{\mathcal {M}}}_{\mathrm {c}}$

The parameter ${\mathcal {M}}$ is large when regular polygonal clusters form because the direction of every particle points toward the center of a regular polygonal cluster such as triangular trimers and rhomboidal tetramers. ${\mathcal {M}}$ becomes large for large *q* and small *l* (Fig. 6d), which agrees with the formation of triangular trimers and rhomboidal tetramers (Figs. 3a, b and  4d). $\sigma _z$ is used as a parameter indicating the three-dimensionality of small clusters because the distribution of the *z*-coordinate of attractive spheres spreads when three-dimensional clusters are created. Therefore, both ${\mathcal {M}}$ and $\sigma _z$ are used to determine the shapes of small clusters. The criteria for classifying cluster types are listed in Table 1. Because three-dimensional chain-like clusters are not observed in the simulations, large clusters for which the size is comparable to *N* are classified into two-dimensional island-like clusters or two-dimensional chain-like clusters. Small clusters are classified into three-dimensional elongated clusters, two-dimensional elongated clusters, or two-dimensional regular clusters. Checks were made as to whether the criteria used for the classification are consistent with snapshots for several sets of *l* and *q*; the criteria were confirmed as reasonable.

**Figure 7:**
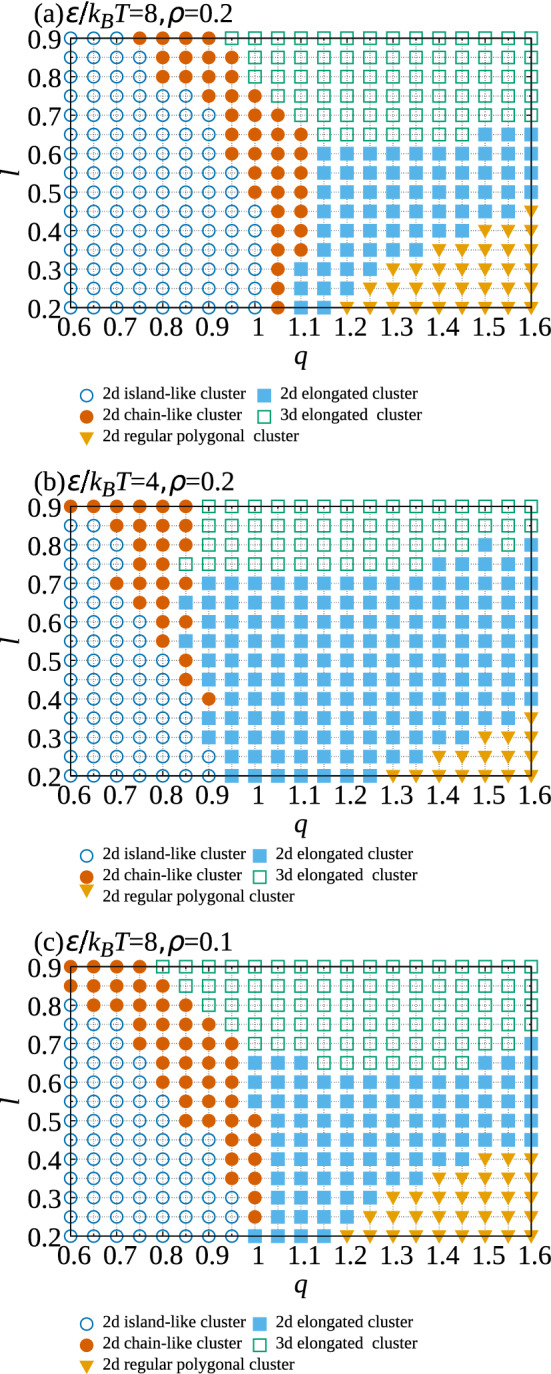
Dependence of cluster type on *q* and *l* for ( $\epsilon /k_{\mathrm {B}}T, \rho )=$ (**a**) (8.0, 0.2), (**b**)(4.0, 0.2), and (**c**) (8.0, 0.1).

Simulations were also performed for $\epsilon /k_{\mathrm {B}}T=4.0$ and $\epsilon /k_{\mathrm {B}}T=8.0$ and analyzed to determine whether the dependence of cluster shape on *l* and *q* changes with temperature. Figure 7 shows how the cluster type depends on *l* and *q* for those temperatures; large island-like clusters form when $q<1$. The parameter regime yielding island-like clusters is much larger than that yielding chain-like clusters. The width of the latter regime becomes small with decreasing *l* because steric hindrance exhibits the same trend. I have already suggested that the formation of three-dimensional double chain-like clusters for which the size is comparable to *N* is possible if $l>\sqrt{3}/2$ for $q=1$. However, the three-dimensional double chain-like clusters were not created in simulations, probably because the temperatures are high in simulations. The chain-like clusters must be easily broken into three-dimensional elongated clusters through thermal fluctuations arising from higher temperatures. Although the upper limit of *l* for forming two-dimensional polygonal clusters increases with increasing *q*, the change in the lower limit of *l* for the formation of three-dimensional elongated clusters is small. Comparing Fig. 7a with Fig. 7b, when the temperature increases, the two limits move to widen the parameter regime associated with two-dimensional clusters. The boundary between the region with large clusters and that with two-dimensional clusters move toward small *q*. Therefore, two-dimensional elongated clusters are created more readily with increasing temperature. The dependence of cluster shapes on $\rho$ is also shown in Fig. 7c. When $\rho$ becomes lower than that in Fig. 7a, the parameter regimes with small clusters hardly change, but the parameter regime with island-like clusters decreases by spreading the parameter regime with two-dimensional chain-like clusters.

## Summary

I performed isothermal-isochoric Monte Carlo simulations in which two parameters *l* and *q* were controlled and results were analyzed to determine the types of clusters formed in thin systems. With $q \le 1$, satisfying that the radius of the attractive area is larger than that of the non-attractive area in the dumbbell-like one-patch particles, large island-like clusters were created. Voids were frequently created in island-like clusters when $\varvec{{\hat{n}}}$ is restricted in the two-dimensional systems^44^. However, the formation of voids in island-like clusters was avoided in thin systems because the three-dimensional rotation of dumbbell-like patchy particles is allowed and $\varvec{{\hat{n}}}$ orientate normal to the flat plane.

With $L_z$ set slightly longer than the long axis of the dumbbell-like patchy particles, the formation of large three-dimensional chain-like cluster seemed possible if *l* was sufficiently large and $\hat{\varvec{n}}$ aligned with the *xy*-plane. However, they did not form and three-dimensional elongated cluster formed instead, probably because of thermal fluctuations. If the temperature is set lower, three-dimensional chain-like clusters may be created because the energy of three-dimensional chain-like clusters is the same as that of island-like clusters.

The significant difference between spherical patchy particles and the dumbbell-like patchy particles is remarkable in the parameter regime with elongated clusters. In the two-dimensional systems with spherical patchy particles, the unit of elongated clusters is a triangular trimer. The elongated clusters are created by the connection of the triangular trimers^7,9,10^. In the three-dimensional systems, large polyhedral clusters such as tetrahedral cluster and octahedral cluster are created^1,4,47^. However, in the systems with dumbbell-like patchy particles, because the shape of particles is isotropic, elongated clusters are more irregular than those observed in the systems with spherical patchy particles, and large polyhedral clusters are not created in the three-dimensional systems.

In our simulations, a triangular lattice was created in island-like clusters because the interaction length was set short, whereas in a previous study the lattice in island-like clusters was square^44^. The difference in interaction length affected the regular polygonal clusters. When the interaction length was long, regular square clusters were created because the particles in the diagonal positions can interact with each other. However, square clusters were not created and rhomboidal clusters formed because the interaction length was sufficiently short. Because the systems were very thin, the cluster types were restricted in the simulations. If the system width were wider^41,47^ clusters and structures which were not observed may be created.

## Methods

In my isothermal-isochoric Monte Carlo simulations, the interaction potential between two dumbbell-like particles is the modified Kern-Frenkel potential^42–44^. For the *i*-th and *j*-th particles, the potential is expressed as $U_{ij}= U^{\mathrm {att}} + U^{\mathrm {rep}}$, $U^{\mathrm {rep}}$ being the hard-core repulsive interaction preventing pairs of particles from overlapping, and $U^{\mathrm {att}}$ an attractive potential given by^42–44^ $U^{\mathrm {att}} = U^{\mathrm {SW}}(r_{ij})f(\varvec{{\hat{r}}}_{ij}, \varvec{{\hat{n}}}_{i}, \varvec{{\hat{n}}}_{j})$. $r_{ij}= |\varvec{r}_{1,j}-\varvec{r}_{1,i}|$, $\hat{\varvec{n}}_{i}=(\varvec{r}_{1,i}-\varvec{r}_{2,i})/d$, and $\varvec{{\hat{r}}}_{ij}=\varvec{r}_{ij}/r_{ij}$, where $\varvec{r}_{1,i}$ and $\varvec{r}_{2,i}$ denote the positions of the centers of the attractive and non-interactive spheres in the *i*-th dumbbell-like particle, respectively. $U^{\mathrm {SW}}(r_{ij})$ is the square-well potential defined as

1 $$\begin{aligned} U^{\mathrm {SW}}(r_{ij}) = {\left\{ \begin{array}{ll} -\epsilon &{} (\sigma _1 \le r_{ij} \le \sigma _1 + \Delta ) \\ 0 &{} (\sigma _1 + \Delta < r_{ij}) \end{array}\right. }, \end{aligned}$$

where $\epsilon$ is the interaction energy and $\Delta$ is the interaction length. Although $\Delta$ was set to $\sigma _1/2$ in previous studies^42–44^, here $\Delta$ is set to $\sigma _1/10$ to ensure the interaction length is smaller than the particle size as indicated from several experiments^7,9^. In $U^{\mathrm {att}}$, $f (\hat{\varvec{r}_{ij}}, \hat{\varvec{n}}_{i}, \hat{\varvec{n}}_{j} )$ represents the anisotropy in the attractive interaction, which is given by

2 $$\begin{aligned} f (\hat{\varvec{r}_{ij}}, \hat{\varvec{n}}_{i}, \hat{\varvec{n}}_{j} ) = {\left\{ \begin{array}{ll} 1 &{} {{(\hat{\varvec{n}}_{i} \cdot \hat{\varvec{r}}_{ij}> \cos \delta {\text { and}} \hat{\varvec{n}}_{j} \cdot \hat{\varvec{r}}_{ji} > \cos \delta })} \\ 0 &{} {\text {otherwise}} \end{array}\right. }. \end{aligned}$$

Initially, the dumbbell-like particles are put in the system at random and moved without the attractive interaction $10^3$ times for each particle. Then, adding the attractive interaction, rotation and translation trials were performed $2 \times 10^6$ times per particle. The maximum values for the rotation angle and translation distance in a Monte Carlo trial were tuned every $10^2 N$ trials to avoid success rates in the Monte Carlo trials begin too low^43^; here, *N* is the number of particles.
